# Comparison of global definitions of metabolic syndrome in early pregnancy among the Rajarata Pregnancy Cohort participants in Sri Lanka

**DOI:** 10.1038/s41598-022-05919-z

**Published:** 2022-02-07

**Authors:** Imasha Upulini Jayasinghe, Thilini Chanchala Agampodi, Ajith Kumara Dissanayake, Shalka Madushan Srimantha, Suneth Buddhika Agampodi

**Affiliations:** 1 Department of Community Medicine, Faculty of Medicine and Allied Sciences, Rajarata University of Sri Lanka, Saliyapura, 50008 Sri Lanka; 2 Department of Gynaecology and Obstetrics, Faculty of Medicine and Allied Sciences, Rajarata University of Sri Lanka, Saliyapura, 50008 Sri Lanka

**Keywords:** Metabolic syndrome, Epidemiology

## Abstract

Metabolic syndrome (MetS) in pregnancy shows epigenetic associations with intergenerational inheritance of metabolic diseases. The presence of different diagnostic criteria influences MetS prevalence estimates. We evaluated MetS and metabolic derangements to determine the utility of its assessment in early pregnancy. A cross-sectional analysis of metabolic derangements in pregnant women with period of gestation (POG) ≤ 12 weeks was done among Rajarata Pregnancy Cohort participants in Sri Lanka. 2682 women with mean age 27.9 year (SD-5.5) and median POG 8.0wk (IQR-3) were analyzed. Mean levels of triglycerides (TG), total cholesterol (TC), high-density-lipoprotein (HDL), low-density-lipoprotein (LDL), fasting plasma glucose, and 2 h oral glucose tolerance test were 87.71 (SD 38.7), 172.2 (SD 34.7), 49.6 (SD 11.5), 122.6 (SD 32.3), 82.2 (SD 12.8) and 120.3 (SD 11.5) respectively. All serum lipids except LDL increase significantly from 6 to 12 weeks, with TG by 23 and TC by 8 units. High MetS prevalence was observed with AHA/NHLBI (n = 150, 5.6%, 95% CI 4.8–6.5) followed by IDF (n = 144, 5.4%, 95% CI 4.6–6.3), NCEP-ATP III (n = 112, 4.2%, 95% CI 3.4–5.0) and WHO (n = 81, 3.0%, 95% CI 2.4–3.7) definitions respectively. Significant difference in prevalence was noted among different sociodemographic characteristics (p < 0.001). Regardless of the criterion used, the change of metabolic parameters in early pregnancy leads to significant differences in prevalence estimates of MetS. The best MetS definition concerning pregnancy outcomes needs to be determined with prospective studies.

## Introduction

Metabolic syndrome (MetS)^1^, is a cluster of metabolic derangements linked with central obesity, which are known to increase the risk of type 2 diabetes mellitus (T2DM) and cardiovascular diseases (CVD)^2–4^. Dysglycemia, hypertension and atherogenic dyslipidemia are the primary metabolic disturbances of concern in MetS^2^. It is also related to nonalcoholic fatty liver disease, prothrombotic and proinflammatory state of the body^5–7^. The global prevalence of MetS in the adult population is around 20–25%, with a higher prevalence among females^4,8^. The prevalence of MetS varies from 3^9^ to 12.4%^10^ in the obstetric population. While the normal pregnancy itself carries a proinflammatory, prothrombotic, hyperlipidemic and insulin-resistant state^11^, superimposition with maternal MetS can predispose intergenerational inheritance of metabolic diseases in progeny^12,13^.

In 1986, David Barker explained that exposure to the adverse intrauterine environment (restricted intrauterine resources) gives rise to metabolic disease in adulthood^14,15^. This implicates developmental plasticity and fetal programming where maternal malnutrition affects fetal organogenesis in early fetal development^16^. At the other end of this spectrum, an over abundant intrauterine environment results in high birth weight of the offspring predisposing him to later life metabolic diseases^17^. The Developmental Origins of Health and Diseases (DOHaD) hypothesis, together with epigenetic mechanisms, explains these associations between early developmental environment on the risk of getting metabolic diseases later in life^18^. Embryonic exposure to a metabolically unbalanced intrauterine environment alters organogenesis, resulting in phenotypic alterations in the progeny. An emerging body of evidence suggests that the altered intrauterine environment can affect the immediate offspring's phenotype and subsequent generations, activating a vicious cycle of obesity being passed on through generations, ‘the transgenerational epigenetic inheritance of obesity”^19^.

The dynamic process of DNA methylation is epigenetically affected by maternal obesity and other associated metabolic derangements of MetS in its intrauterine environment^19–21^. Recent evidence suggests that maternal glycemic levels, pre-pregnancy Body Mass Index (BMI), and gestational weight gain are associated significantly with altered placental DNA methylation at several points like in genes for adiponectin, leptin, and glucose transporters^22–24^. Adipose tissue and adipokines have a vital role in metabolic homeostasis. Its dysregulation associated with obesity has a detrimental impact on energy metabolism, which lays the background for MetS and its associated metabolic derangements even among the progeny^25^. The obesity driven meta inflammation in pregnant women creates a dysfunctional fetoplacental unit with metabolic, inflammatory and vascular abnormalities^26^, results in adverse maternal and neonatal outcomes by epigenetically affecting the gene expression in the offspring^15^. In MetS pathogenesis, there is reduced adiponectin level and increased leptin level, which are mediated by increased visceral adiposity^25,27^. When a woman with high BMI enters pregnancy, she already has imbalanced adiponectin and leptin levels in the early prenatal period and it directly affects fetal metabolic programming resulting in increased birth weight and future growth trajectories towards obesity in the offspring^15,27,28^.

However, the diagnosis of MetS is still controversial due to the presence of many definitions for diagnosis. The use of different combinations of metabolic derangements as the criteria hence affects the prevalence estimates on MetS^29,30^. Various definitions for the diagnosis of MetS were forwarded by World Health organization—1998 (WHO), National Cholesterol Education Programme Adult Treatment Panel III (NCEP ATP III), American Heart Association/National Heart, Lung and Blood Institute (AHA/NHLBI) and International Diabetes Federation (IDF)^2,4,31–33^, while last two are the most commonly used currently. Lack of consensus on a definition for MetS diagnosis creates a significant gap in its assessment, especially during pregnancy. In this context, the research of MetS in pregnancy is gaining traction, and its impact on early, mid, and late pregnancy, as well as the impact of particular metabolic abnormalities identified in MetS on pregnancy, is attracting scholarly attention. Therefore, evaluation of early pregnancy MetS by several definitions in a larger population is necessary. In Sri Lanka, the prevalence of MetS was reported as 24.3% in all adults according to IDF criteria with a higher prevalence among females and increasing age which match the global trend^34^. Furthermore, due to the high prevalence of abdominal obesity among Sri Lankan females^30^, they are susceptible to the metabolic abnormalities associated with obesity identified in MetS. However, there is a scarcity of research evidence on MetS among Sri Lankan women and during pregnancy. The aim of our study was to determine the prevalence of MetS according to different definitions and to determine the prevalence of individual metabolic derangements of MetS among pregnant women in Anuradhapura district of North Central province of Sri Lanka.

## Methods

**Figure 1:**
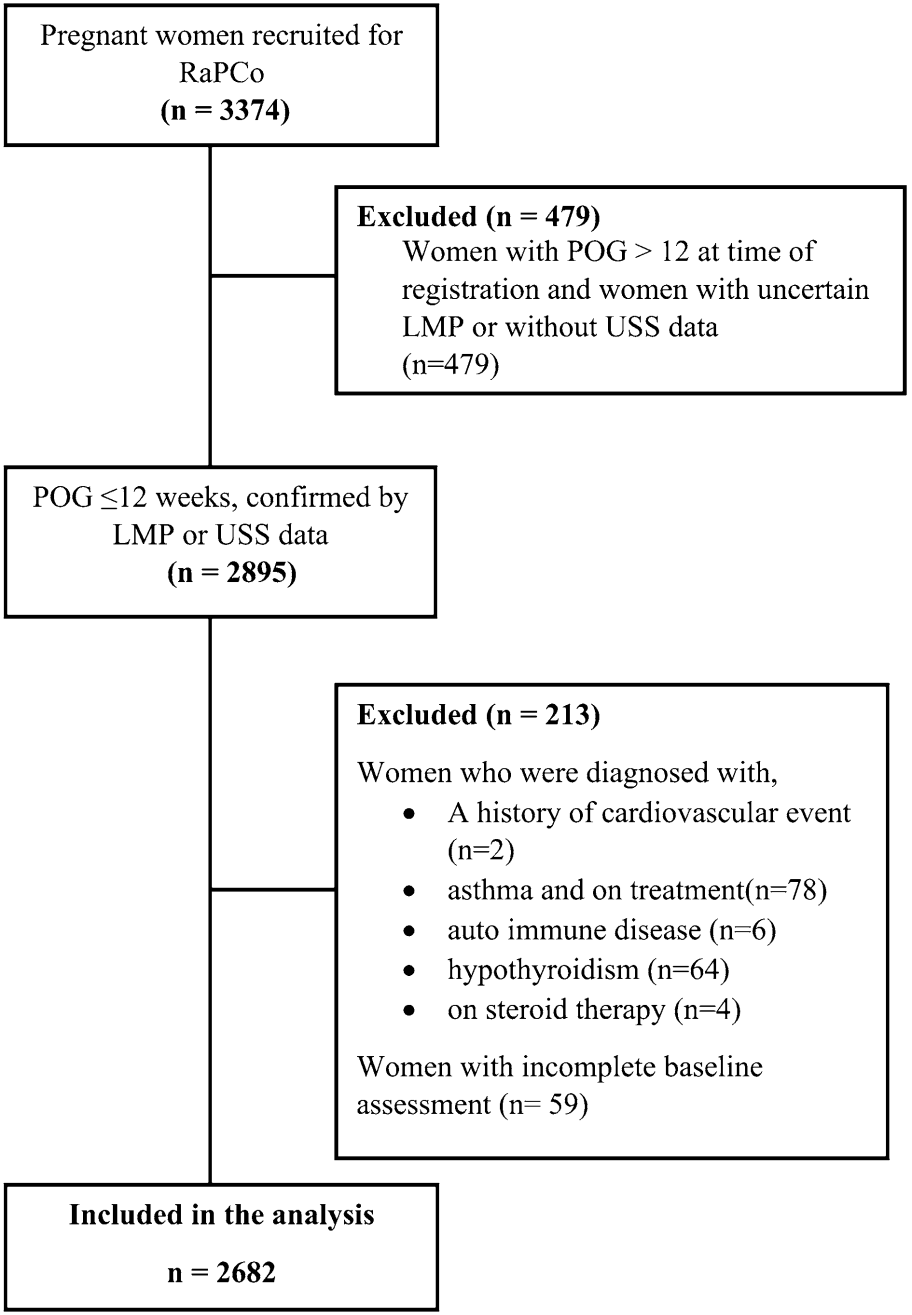
Flow chart of inclusion and exclusion criteria of participants for the analysis.

This study includes the detailed baseline metabolic assessment of the largest maternal cohort in Sri Lanka, the Rajarata Pregnancy Cohort (RaPCo). Methods in detail of the original cohort study is published elsewhere^35^. and there was no involvement of patient and public in designing or conducting the study. The study was conducted in Anuradhapura district, Sri Lanka from 01st of July 2019 to 30th of September 2019. Newly registered pregnant women in the field antenatal clinics during this period were invited for the study. Their period of gestation (POG) was determined using the ultra sound scan (USS) data and for those who are without USS data, with the last menstrual period (LMP). Figure 1 shows how the participants were included for this analysis.

### Data collection

Data was collected in 226 field clinics over 71 days by a team of investigators fully trained on study data collection, including trained medical undergraduates, medical doctors and a nursing officer attended each clinic for data collection. All participants were advised to come with 8–10 h fasting. Every mother who attended the clinic was given an information leaflet with details of the study, blood and data collection procedures. Following detailed explanation, informed written consent was obtained. Pre-tested interviewer administered questionnaire was used for data collection on sociodemographic, gynecological and medical backgrounds. Interviewers were pre trained for the questionnaire with a common questionnaire guideline.

### Anthropometric measurements

Height and weight were measured using portable stadiometer and digital weighing scale respectively according to standard protocol^36^. Waist circumference (WC) was measured with a non-stretchable, calibrated tape placed parallel to the ground and around the waist at mid-way between the lower rib margin and the iliac crest at the end of normal expiration. Hip circumference (HC) was measured at widest part of the buttocks by placing a non-stretchable tape parallel to the ground. Two measurements were taken for each WC and HC and mean value was recorded. All measurements were recorded in standard international units to the nearest 0.1 cm for height, WC, HC and nearest 100 g for weight. BMI and waist hip ratio (WHR) were calculated with the anthropometric data obtained.

### Clinical examination

Every mother was subjected to a clinical examination by medically qualified (MBBS) research team members. Blood pressure was measured using a digital blood pressure meter (OMRON HEM-7320). Each participant was positioned according to the standard protocol for blood pressure measurement. Two readings were taken and minimum values of systolic and diastolic blood pressure each was documented as the blood pressure of the participant.

### Biochemical procedures

A qualified nursing officer conducted venipuncture under universal precautions. 2.5 mL of whole blood was collected into each Sodium fluoride/Potassium oxalate (NaF/K_2_C_2_O_4_) tube for fasting plasma glucose (FPG) and plain tube for serum analysis for lipid profile. Following fasting blood collection, every mother except those with pre diagnosed diabetes mellitus, were given 75 g of glucose dissolved in 300 ml of water and second venipuncture was done after 2 h to collect 2.5 mL of whole blood to NaF/K_2_C_2_O_4_ tube for 2 h oral glucose tolerance test (2 h OGTT). All the samples were labelled with serial code number and transported in a cool box within 4 h of collection to Public Health Research Laboratory of Faculty of Medicine and Allied Sciences, Saliyapura. All the samples were analyzed on the same day by an automated clinical chemistry analyzer (Mindray BS-240). In cases of a delay in analyzing the samples for tests other than plasma glucose, (for less than 3 days), the serum and plasma samples were stored in a refrigerator in 4 °C. If the delay would be more than three days, samples were immediately frozen at -70 °C. Investigation results of FPG, 2 h OGTT, high-density lipo protein (HDL), low-density lipo protein (LDL), triglycerides (TG), total cholesterol (TC) were recorded in standard international units.

### Definitions

For this study, MetS was defined according to four commonly used definitions for the comparison purpose (Box 1). BMI evaluation was done using Asia Pacific Guidelines^36^ and classified as underweight (< 18.5 kg m^−2^), normal (< 18.5–22.9 kg m^−2^), pre-obese (23–24.9 kg m^−2^), obese class I (25–29.9 kg m^−2^) and obese class II (≥ 30 kg m^−2^). In diagnosing MetS by IDF criteria and AHA/NHLBI criteria, WC ≥ 80 cm was used as the ethnic specific cutoff value. Mother reported, records available cases were taken as previously diagnosed mothers with chronic hypertension andT2DM.

A POG until completion of 12 weeks was considered as the “first trimester” for the study. For data analysis, participants were categorized into 4 categories according to their POG at the time of registration and data collection. (1. POG 4–6 weeks, 2. POG 7–8 weeks, 3. POG 9–10 weeks, 4. POG 11–12 weeks). Further analysis of MetS among the participants was continued using IDF criteria.

Box 1: The definitions of metabolic syndrome used in this study **Definitions of Metabolic Syndrome**

*1. International Diabetes Federation (IDF) criteria*

Central obesity (defined as waist circumference with ethnicity specific values) plus any two of the following,

- Raised triglycerides ≥ 150 mg/dL (1.7 mmol/L) or specific treatment for this lipid abnormality
- Reduced HDL cholesterol < 50 mg/dL or specific treatment for this lipid abnormality
- Raised blood pressure ≥ 130/85 mm Hg or treatment of chronic hypertension
- Raised fasting plasma glucose ≥ 100 mg/dL (5.6 mmol/L), or previously diagnosed type 2 diabetes mellitus

*2. American Heart Association/National Heart, Lung and Blood Institute (AHA/NHLBI) criteria*

Any three of the following,

- Elevated waist circumference (according to population and country specific definitions)
- Plasma triglycerides ≥ 150 mg/dl or specific treatment for this lipid abnormality
- HDL cholesterol < 50 mg/dl or specific treatment for this lipid abnormality
- Blood pressure ≥ 130/85 mmHg or treatment of previously diagnosed hypertension
- Fasting plasma glucose ≥ 100 mg/dl or on drug treatment for elevated glucose

*3. National Cholesterol Education Programme Adult Treatment Panel III (NCEP ATP III) criteria*

Any three of the following,

- Waist circumference > 88 cm
- Plasma triglycerides > 150 mg/dl
- HDL cholesterol < 50 mg/dl
- Blood pressure ≥ 130/85 mmHg
- Fasting plasma glucose ≥ 110 mg/dl

*4. World Health Organization (WHO) 1998 criteria*

Presence of insulin resistance or type 2 diabetes mellitus (T2DM) or impaired fasting glucose (IFG) or impaired glucose tolerance (IGT) plus any two of the following,

- Raised arterial pressure ≥ 140/90 mmHg
- Raised plasma triglycerides ≥ 150 mg/dl
- Low HDL cholesterol < 39 mg/dl
- Central obesity (BMI > 30 kg m^−2^ and/or waist to hip ratio > 0.85)
- Urinary albumin excretion rate > 20 µg min^−1^ or albumin creatinine ratio > 30 mg/g

### Data quality and analysis

Data collecting members and principal investigators had weekly meetings for internal review of process and procedures in data collection, entry and storage. The data collectors were frequently supervised and feedback given at field data collection. The equipment was regularly calibrated and checked for technical errors. External quality control was carried out regularly for automated analyzer every fortnight. All data entry was random checked and entered according to serial number coding into the data base on the same day and any missing data were recorded uniformly. Data analysis was done using IBM Statistical Package for the Social Sciences (SPSS) version 26. Descriptive prevalence data was calculated and presented as proportions and percentages with 95% confidence intervals (CI). All the mean values were presented with standard deviation (SD) or standard error of mean (SEM). One way ANOVA statistical testing was done to compare the differences in means of biochemical parameters among different POG categories. Statistical significance was reported to the significance value of < 0.05.

Ethical clearance for the study was obtained from the Ethics Review Committee of Faculty of Medicine and Allied Sciences, Rajarata University of Sri Lanka (ERC/2019/07). The study was conducted in accordance with the rules and guidelines of the ethical approval obtained.

## Results

Altogether, 2682 first trimester pregnant women were included for this analysis. Their mean age was 27.9 years (SD 5.5) with a range from 15 to 44 years. The sociodemographic characteristics of the study sample are given in Table 1. Mean and median POG of the study participants was 8.4 weeks (SD 1.8) and 8.0 weeks (IQR 3) respectively. There were 41 (1.5%), 71 (2.6%), 31 (1.2%) women in the study sample who reported (with available documents) a past medical history of T2DM, hypertension and dyslipidemia respectively. Out of 2682 women, 431 (16.1%) claimed that they had irregular menstruation.

**Table 1:** Characteristics of the study population.

Characteristic	n	%
**Ethnicity**		
Sinhala	2360	88.0
Moor	287	10.7
Other	35	1.3
**Age at conception (years)**		
< 20	190	7.1
20–24	542	20.2
25–29	950	35.4
30–34	647	24.1
35–39	303	11.3
40–44	50	1.9
**Highest level of education**		
Up to grade 10	281	10.6
GCE Ordinary Level	1307	49.3
Grade 12–13	1063	40.1
**Marital status**		
Yes	2633	98.2
No	49	1.8
**Gravidity**		
1	829	30.9
2	856	31.9
3	661	24.7
4	242	9.0
5 or more	92	3.4
**Parity**		
0	929	34.6
1	996	37.1
2	630	23.5
3	107	4.0
4	18	0.7
**POG at the time of recruitment (weeks)**		
4	26	1.0
5	104	3.9
6	290	10.8
7	494	18.4
8	607	22.6
9	455	17.0
10	303	11.3
11	257	9.6
12	146	5.4

*GCE* general certificate of education, *POG* period of gestation.

The summary of anthropometric measurements and biochemical parameters of the participants are shown in Table 2. Even though the mean WC is below the ethnic specific cut off value, the mean WHR is in the range of “moderate risk” for future cardiovascular adverse events. Based on WHR, 37.4% (n = 1002) were at high risk and 29.5% (n = 791) were at moderate risk for cardiovascular events in the future. Further classification of BMI showed 417 (15.5%), 663 (24.7%) and 259 (9.7%) pregnant women in respective classes of pre-obese, obese class I and obese class II. 33.0% (n = 884) of the study sample was in the normal BMI range (18.5–22.9 kg m^−2^).

**Table 2:** Anthropometric data, blood biochemistry and physical measures of 2682 first trimester pregnant women.

	Mean	SD	SEM
**Anthropometric measurements**			
Weight (kg)	55.4	11.9	0.2
Height (cm)	154.1	5.7	0.1
Waist circumference (cm)	76.5	11.6	0.2
Hip circumference (cm)	91.6	10.1	0.2
Waist hip ratio	0.83	0.07	0.00
Body mass index	23.32	4.79	0.09
**Biochemical measurements**			
Triglycerides (mg/dL)	87.71	38.7	0.76
Total cholesterol (mg/dL)	172.2	34.7	0.68
High density lipoproteins (mg/dL)	49.6	11.5	0.23
Low density lipoproteins (mg/dL)	122.6	32.3	0.64
Fasting plasma glucose (mg/dL)	82.2	12.8	0.25
2 h oral glucose tolerance test (mg/dL)	120.3	33.6	0.68
**Physical measurements**			
Systolic blood pressure (mmHg)	102.7	11.5	0.22
Diastolic blood pressure (mmHg)	65.4	8.2	0.16

*SD* standard deviation, *SEM* standard error of mean.

Analysis of biochemical measures among POG categories shows that there is an increase in the mean values of serum lipids over the weeks of gestation (Table 3) but a similar pattern was not evident with the serum glucose values and blood pressure measurements. The change of all biochemical parameters within POG categories was significant except for the changes in LDL and blood pressure.

**Table 3:** Change of biochemical parameters and blood pressure values among POG categories among 2682 first trimester pregnant women.

	POG categories								F	Sig.*
	4–6 weeks		7–8 weeks		9–10 weeks		11–12 weeks			
	Mean	SD	Mean	SD	Mean	SD	Mean	SD		
TG	78.42	1.91	83.38	1.17	90.15	1.53	101.19	2.36	27.33	0.000
TC	167	1.80	171.29	1.12	173.80	1.35	175.92	2.04	4.89	0.002
HDL	48.02	0.56	48.87	0.37	50.66	0.47	51.13	0.67	8.26	0.000
LDL	118.97	1.61	122.42	1.06	123.14	1.24	124.78	1.87	2.02	0.109
FPG	82.10	0.52	82.68	0.42	81.23	0.41	79.44	0.58	5.79	0.001
2 h OGTT	116.11	1.66	122.58	1.21	120.13	1.24	118.92	1.72	3.35	0.018
SBP	103.17	0.55	102.91	0.38	102.48	0.46	101.73	0.65	0.984	0.399
DBP	65.21	0.40	65.58	0.28	65.54	0.33	64.51	0.44	1.58	0.193

*POG* period of gestation, *SD* standard deviation, *TG* triglycerides, *TC* total cholesterol, *HDL* high density lipoproteins, *LDL* low density lipoproteins, *FPG* fasting plasma glucose, 2 h *OGTT* 2 hour oral glucose tolerance test, *SBP* systolic blood pressure, *DBP* diastolic blood pressure.

*Significance testing was done using one way ANOVA. P value < 0.05 was taken as the level of significance.

Of the study sample, 18.8% (n = 504) had high TC (≥ 200 mg/dl) value and 70.4% (n = 1889) had high LDL value (≥ 100 mg/dl). Lipid abnormalities were the commonest metabolic derangements observed, with high LDL as the commonest followed by low HDL, high TC and high TG respectively. Also, 242 (9.4%) and 31 (1.2%) women had FPG values of 92–125 mg/dl and ≥ 126 mg/dl respectively. There were 236 (9.6%) women with 2 h OGTT value of 153–199 mg/dl and 61 (2.5%) women with 2 h OGTT value ≥ 200 mg/dl.

Table 4 summarizes the prevalence of each metabolic derangements as described in each criterion for metabolic syndrome (MetS). In each criterion of MetS except WHO criteria, the commonest metabolic derangement was low HDL followed by high LDL, high TG, high FPG and low BP respectively. In WHO criteria, the commonest abnormality was high LDL.

**Table 4:** Prevalence of metabolic derangements (parameters) among a population-based cohort of 2682 pregnant women with period of gestation < 12 weeks from Anuradhapura, Sri Lanka.

Metabolic derangement		Number fulfilling the specific criteria							
		IDF		AHA/NHLBI		NCEP-ATP III		WHO	
	N	n	%	n	%	n	%	n	%
**Waist circumference**									
≥ 80 cm^e,f^	2682	1005	37.5	1005	37.5				
≥ 88 cm^g^	2682					464	17.3		
**Plasma glucose**									
FPG ≥ 100^e,f^	2682			109	4.1				
	1005^c^	62	43.1						
FPG ≥ 110^ g^	2682					66	2.5		
Insulin resistance^a,h^	2682							544	20.3
**Serum lipids**									
TG ≥ 150^e,f,g,h^	2682			185	6.9	185	6.9		
	1005	76	52.8						
	544^d^							67	12.3
HDL < 50^e,f,g^	2682			1409	52.5	1409	52.5		
	1005	134	93.1						
HDL < 39^ h^	544							107	19.7
**Blood pressure**									
≥ 130/85^e,f,g^	2682			62	2.3	62	2.3		
	1005	26	18.1						
≥ 140/90^ h^	544							7	1.3
**Central obesity**^**b,h**^	544							293	53.9

^a^Insulin resistance- identified by any one of these: a) Type 2 diabetes mellitus b) Impaired fasting glucose c) Impaired glucose tolerance.

^b^BMI >  = 30 and/or WHR > 0.85.

*FPG* fasting plasma glucose, *TG* triglycerides, *HDL* high density lipoproteins, *BMI* body mass index, *WHR* waist hip ratio.

N-Shows the total number of samples considered for each metabolic derangement.

^c^1005 is the number of women with ≥ 80 cm waist circumference.

^d^544 is the number of women with insulin resistance under WHO criteria.

^e^*IDF* International Diabetes Federation.

^f^*AHA/NHLBI* American Heart Association/National Heart, Lung and Blood Institute.

^g^*NCEP ATP III* National Cholesterol Education Programme Adult Treatment Panel III.

^h^*WHO* World Health organization.

Table 5 shows the prevalence of MetS by AHA/NHLBI, IDF, NCEP ATP III criteria and its distribution by socio-demographic characteristics. The prevalence of MetS by WHO criteria was 4.2% (n = 112, 95% CI 3.4–5.0). Further analysis shows that there is a significant difference in prevalence of MetS among different age categories, ethnic groups and gravida of women (p < 0.001).

**Table 5:** Prevalence of metabolic syndrome among first trimester pregnant women in Anuradhapura district and its distribution by socio-demographic characteristics in the study sample.

	AHA/NHLBI criteria			IDF criteria			NCEP ATP III criteria		
	n	%	95% CI	n	%	95% CI	n	%	95% CI
*Prevalence of metabolic syndrome*	150	5.6	4.8–6.5	144	5.4	4.6–6.3	81	3.0	2.4–3.7
**Age**									
< 20	4	2.11	0.58–5.30	4	2.11	0.58–5.30	3	1.58	0.54–4.54
20–24	16	2.95	1.70–4.75	15	2.77	1.56–4.52	8	1.48	0.75–2.89
25–29	36	3.79	2.75–5.20	35	3.68	2.66–5.08	22	2.32	1.53–3.48
30–34	51	7.88	5.93–10.23	49	7.57	5.66–9.89	25	3.86	2.63–5.64
35–39	36	11.88	8.71–16.01	35	11.55	8.42–15.64	19	6.27	4.05–9.59
40–44	7	14	5.82–26.74	6	12.0	4.53–24.31	4	8	3.15–18.84
**Ethnicity**									
Sinhala	111	4.7	3.92–5.63	106	4.49	3.73–5.40	56	2.37	1.83–3.07
Moor	34	11.84	8.35–16.16	33	11.49	8.05–15.77	21	7.31	4.84–10.93
Other	5	14.28	6.26–29.38	5	14.28	6.26–29.38	4	11.43	4.54–25.95
**Gravidity**									
1	30	3.62	2.45–5.13	29	3.50	2.36–4.99	18	2.17	1.38–3.41
2	46	5.37	4.05–7.09	44	5.14	3.85–6.83	23	2.69	1.80–4.00
3	40	6.05	4.48–8.14	39	5.90	4.35–7.96	19	2.87	1.85–4.45
4	22	9.09	6.08–13.38	20	8.26	5.41–12.42	13	5.37	3.17–8.97
≥ 5	12	13.04	7.62–21.43	12	13.04	7.62–21.43	8	8.7	4.47–16.23
**Education level**									
Up to grade 10	26	9.25	6.39–13.21	24	8.54	5.81–12.39	17	6.05	3.81–9.47
GCE O/L	63	4.82	3.79–6.12	63	4.82	3.79–6.12	36	2.75	2.00–3.79
Grade 12–13	57	5.36	4.16–6.88	53	4.99	3.83–6.46	28	2.63	1.83–3.78

*IDF* International Diabetes Federation, *AHA/NHLBI* American Heart Association/National Heart, Lung and Blood Institute, *NCEP ATP III* National Cholesterol Education Programme Adult Treatment panel III,  *CI* confidence interval, *GCE O/L* general certificate of education ordinary level.

**Figure 2:**
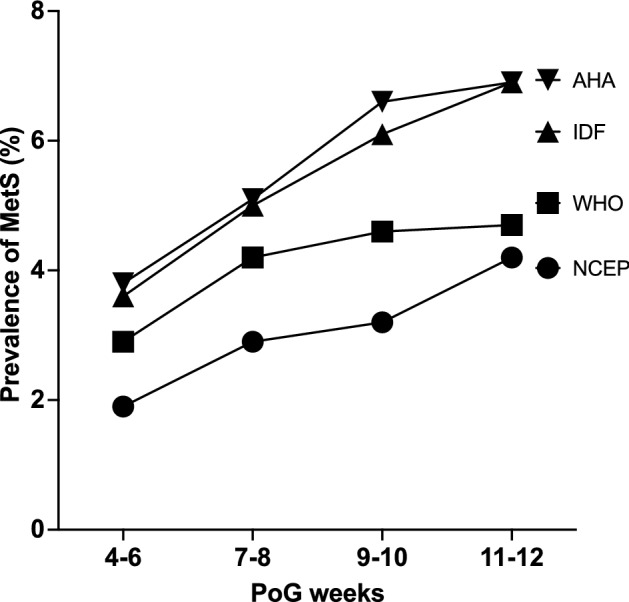
Prevalence of metabolic syndrome by each definition within categories of the period of gestation (POG) (*AHA/NHLBI* American Heart Association/National Heart, Lung and Blood Institute, *IDF* International Diabetes Federation, *WHO* World Health organization, *NCEP ATP III* National Cholesterol Education Programme Adult Treatment Panel III).

The prevalence of MetS increases steadily with increasing POG (Fig. 2). However, the observed difference in prevalence within POG categories was not significant (p > 0.05).

## Discussion

Using a large population based maternal cohort, we found a 5.4% (CI 4.6–6.3) prevalence of MetS (according to IDF criteria) among pregnant women in rural Sri Lanka. The prevalence of metabolic parameters across different POGs in early pregnancy were calculated and was found to increase significantly with the advancement of pregnancy even during the first trimester. The novelty and importance of this study is represented by capturing a district representative sample of pregnant women in very early in their pregnancy and use of standard rigorous techniques in obtaining the measurements. Our findings add to large-scale estimates of MetS prevalence among pregnant women in a South Asian population.

Our results show that both IDF criteria and AHA/NHLBI criteria capture a higher number of cases with MetS in pregnancy. This finding is consistent with findings from a study on the prevalence of MetS in Sri Lankan females, which found a greater age-adjusted prevalence of MetS when the IDF definition is used instead of the NCEP ATP III criteria^34^. However, our findings contradict with those of another research among urban Sri Lankans, which found a higher prevalence using NCEP ATP III criteria^30^. None of these studies, unlike ours, examined MetS using AHA/NHLBI criteria, making comparisons of MetS prevalence estimates even more difficult. Despite this, there are no national and regional level evidence figures on the prevalence of MetS among pregnant women in their early first trimester. A cross country, multi-center cohort study (SCOPE study) done between 2004 and 2011 among pregnant women showed a 12.3% prevalence of MetS in early pregnancy when IDF criteria was used^37^. However this study explains the prevalence estimates of a non-Asian community, where ethnic diversity should be a worry for Asians to make statistical inferences. The SCOPE study included only nulliparous women, and initial anthropometric and biochemical evaluation were done at 15 ± 1 week gestation. The women in our study were both nulliparous and multiparous, and they were in the early stages of the first trimester, with a median POG of 8 weeks (IQR 3), and all biochemical and anthropometric assessments were completed before the completion of 12 weeks of POG. This early metabolic examination in pregnant women is critical for identifying individuals who already have metabolic abnormalities and unfavorable anthropometric measurements prior to pregnancy, with or without pregnancy consequences.

Our results also showed a significant change in lipid levels over the gestational period we studied, and hence evaluation of these biochemical values late in the gestation would have masked effects on biochemical profile by pregnancy itself. Even though low serum HDL is the concern for MetS, its level also has a rising trend over the gestation period towards 12 weeks of gestation. This pattern of lipid changes is keeping in with the normal pregnancy physiology^38^. In our total study, the commonest metabolic derangement among pregnant women was lipid abnormalities. High LDL level was the commonest followed by low HDL level. However, among the pregnant women with MetS, low HDL level was the commonest, followed by high LDL level and high TG level, respectively. This atherogenic dyslipidemia is similar to the commonly found lipid abnormalities among South Asians^39,40^. This pattern was also evident in a recent study on early pregnancy gestational lipid profile among a group of women in Netherland^41^. A recent study in United States revealed that low HDL level in early pregnancy affects DNA methylation and associated with accelerated placental ageing^42^.

In contrast to above finding, hypertriglyceridemia was the most common lipid abnormality among participants in SCOPE study. In Amsterdam born children and their development (ABCD) study in 2004, the researchers showed that early pregnancy hypertriglyceridemia was associated with pregnancy induced hypertension, preeclampsia, preterm birth and large for gestational age babies^43^. Also, an emerging body of evidence suggests that atherogenic dyslipidemia in MetS in early pregnancy is known to be associated with various adverse pregnancy outcomes^44–47^. In our study, other than the significant prevalence of hypertriglyceridemia, a significant increase in TG’s mean values (by 23 units) from 6 to 12 weeks of gestation was observed. Our study indicates a strong baseline assessment to generate evidence with this regard for South Asian population.

Dysglycemia with insulin resistance is another key feature in MetS. In our population, AHA/NHLBI captured a higher number of women with dysglycemia compared to IDF criteria, but those with insulin resistance as described by WHO criteria were the highest number with dysglycemia. The prevalence estimates for dysglycemia varies depending on the criteria, which has a major influence on the overall prevalence of MetS. Even though there is an 83.9% prevalence of raised fasting glucose among the SCOPE study participants, it was evident in only 43.1% of our population. According to WHO’s recent recommendations on hyperglycemia first detected in pregnancy^48^, 15.9% of our pregnant women had GDM and 3.2% had DM (which was preconceptionally undiagnosed) in their first trimester of pregnancy. This hyperglycemia in pregnancy is underestimated since we have not measured 1-h plasma glucose value for OGTT and random plasma glucose value. A systematic review and meta-analysis by Logan et al. in 2014 has shown a positive relation between maternal diabetes and infant adiposity^49^. A group of researchers in South Carolina recently gave evidence that in the background of maternal hyperglycemia with high LDL level, placental barrier function and angiogenicity of trophoblasts are impaired leading to preeclampsia^50^. In our study population also, 292 and 62 pregnant women with GDM and DM respectively, had serum level of LDL > 100 mg/dl in the first trimester.

Among the recruited participants for our study, the prevalence patterns of overweight and obesity was similar to those detected by the national level noncommunicable disease risk factor survey in Sri Lanka in 2015^51^. This survey detected a mean BMI value of 23.5 kg m^−2^ (95% CI 23.2–23.7) among females, which is also similar to the mean BMI value detected in our population. The results on anthropometric measures of central obesity show that many of our study participants were affected with overweight/obesity. Those who had MetS had higher mean values for BMI (obese class I), WHR (high risk) and also for all other metabolic parameters measured.

The Healthy Start Study in Colarado showed that maternal BMI is independently and positively associated with neonatal adiposity^52^. A recent Taiwanese study among pregnant women shows that early pregnancy overweight/obesity cluster metabolic risk factors and result in adverse pregnancy outcomes^53^. Because central obesity is a critical characteristic in MetS, a measure that is ethnically appropriate should be included in the MetS diagnosis criteria. This is corroborated by our observation that the IDF and AHA/NHLBI criteria, which employ ethnic-specific anthropometric measurements, had a greater prevalence of MetS than the WHO and NCEP ATP III criteria. There are several limitations of our study. USS data at the time of recruitment, was available in only 59.5% of the participants. POG calculation was therefore based on LMP in 40.5% of participants. Measurement of urinary albumin excretion and insulin resistance was not done in our study, which may underestimate MetS using WHO definition. Participants who claimed to be diagnosed with a lipid abnormality, but the specific lipid abnormality was not known or not documented, were not included under IDF and AHA/NHLBI classifications leading to a probable underdiagnosis.

In conclusion, our data clearly shows that irrespective of the criterion used for diagnosis, MetS is common even in young pregnant women in rural Asian settings. More importantly we showed the rapid change of parameters used for the diagnosis of MetS within the first few weeks of pregnancy, which can lead to significant differences in the prevalence estimates of MetS in pregnancy. Since the prevalence of MetS based on different definitions vary, the most appropriate definition predicting adverse pregnancy outcomes needs to be investigated using prospective designs with planned outcome assessments. In countries where the cardiovascular risk assessment and screening are still in infancy, early pregnancy metabolic assessment will be an opportunistic screening with high population coverage.

## Data Availability

The data that support the findings of this study are available from the corresponding author upon reasonable request.
